# The prognostic factors for patients with hematological malignancies admitted to the intensive care unit

**DOI:** 10.1186/s40064-016-3714-z

**Published:** 2016-11-29

**Authors:** Qian Cheng, Yishu Tang, Qing Yang, Erhua Wang, Jing Liu, Xin Li

**Affiliations:** 1 Department of Hematology, The Third Xiangya Hospital, Central South University, Changsha, 410013 Hunan China; 2 Department of Medicine, Yale New Haven Hospital, New Haven, CT USA

**Keywords:** Intensive care, Hematological malignancies, Prognostic factors, Asian countries

## Abstract

Owing to the nature of acute illness and adverse effects derived from intensive chemotherapy, patients with hematological malignancies (HM) who are admitted to the Intensive Care Unit (ICU) often present with poor prognosis. However, with advances in life-sustaining therapies and close collaborations between hematologists and intensive care specialists, the prognosis for these patients has improved substantially. Many studies from different countries have examined the prognostic factors of these critically ill HM patients. However, there has not been an up-to-date review on this subject, and very few studies have focused on the prognosis of patients with HM admitted to the ICU in Asian countries. Herein, we aim to explore the current situation and prognostic factors in patients with HM admitted to ICU, mainly focusing on studies published in the last 10 years.

## Background

The triage and management of patients with hematological malignancies (HM) in life-threatening situations presents a paradox for clinicians (Hill 2010). Historical research established more than 25 years ago found that HM patients admitted to the intensive care unit (ICU) have poor prognosis due to their acute illness and adverse effects resulting from intensive chemotherapy (Lloyd-Thomas et al. 1988; Yau et al. 1991; Brunet et al. 1990). Undeniably, recent advances in life-sustaining therapies and close collaborations between hematologists and intensive care specialists have resulted in a paradigm shift and in the improvement of the prognosis of patients with HM admitted to the ICU (Hampshire et al. 2009; Azoulay et al. 2013; Bird et al. 2012; Hill et al. 2012). In Western countries, the ICU mortality rate of patients with HM has abated from 65 to 80% in the 1980s (Lloyd-Thomas et al. 1988; Yau et al. 1991; Schuster and Marion 1983) to 34–56% today (Hampshire et al. 2009; Bird et al. 2012; Geerse et al. 2011). A large prospective trial performed by Azoulay et al. (2013) noted that the hospital, day-90, and 1-year survival rates for patients with HM admitted to ICU were 60.7, 52.5, and 43.3%, respectively. Additionally, while many studies from different countries have examined the prognostic factors of these critically ill HM patients, the relative significance of many factors remains controversial, as many of them have not been confirmed in multicenter prospective studies (Hampshire et al. 2009; Lamia et al. 2006; Gordon et al. 2005; Evison et al. 2001; Benoit et al. 2003; Liu et al. 2015). To the best of our knowledge, there has been no up-to-date review on this subject since 2010 (Hill 2010). Accordingly, we might ask, has the value of some classical prognostic factors changed? Furthermore, most of the earlier studies were mainly focused on Western countries, while an increasing number of Asian countries have published studies that have shown different results regarding this subgroup of patients. For example, in some retrospective study in China (Evison et al. 2001; Benoit et al. 2003; Liu et al. 2015), HM patients had higher mortality rate and less access to the ICU, as compared to reports published by Western countries (Azoulay et al. 2013; Bird et al. 2012; Merz et al. 2008). Considering this point of view, whether Asian countries will compare with each other remains elusive, and it is unclear what factors might contribute to the different prognosis observed in these countries. We aim to explore these questions in this review article, with a particular focus on studies published in the last 10 years.

## Clinical factors

### Factors predictive of prognosis for HM patients admitted to the ICU

#### Use of mechanical ventilation

Most studies have shown that acute respiratory failure is one of the primary reasons for ICU admission for critically ill HM patients (Bird et al. 2012; Geerse et al. 2011; Merz et al. 2008). Generally, there are three ways to improve respiratory failure: supplemental oxygen (e.g., nasal cannula or face mask), invasive mechanical ventilation (IMV, e.g., via an endotracheal tube or tracheostomy), or noninvasive mechanical ventilation (NIMV, e.g., bilevel or continuous positive airway pressure). A large retrospective study in 2000 was the first to identify IMV as strong prognostic factor in critically ill patients with HM (Staudinger et al. 2000), and this was later proved to be the case in nearly all studies (Lloyd-Thomas et al. 1988; Geerse et al. 2011; Lamia et al. 2006; Evison et al. 2001; Turkoglu et al. 2011; Kroschinsky et al. 2002; Owczuk et al. 2005; Rabbat et al. 2005; Namendys-Silva et al. 2013; Yeo et al. 2012). Generally, more than half of the critically ill HM patients need IMV to improve respiratory failure, and most of them eventually died in the hospital or ICU (Geerse et al. 2011; Liu 2015; Turkoglu et al. 2011). An explanation for the high mortality rate in this subgroup of patients may attribute to the co-occurrence of severe pulmonary dysfunction and nosocomial infections, such as ventilator-associated pneumonia (Kwak et al. 2010). The results of previous studies also showed that noninvasive mechanical ventilation could significantly reduce the rates of endotracheal intubation and serious complications, and thus plays a protective role, especially in immunosuppressed hosts (Hilbert et al. 2001a; Azoulay et al. 2001). Moreover, the improvement in NIMV significantly decreases the overall mortality rate of critically ill patients with HM (Liu et al. 2015; Belenguer-Muncharaz et al. 2013). In a multicenter, ten-year retrospective observational study, Belenguer-Muncharaz et al. (2013) compared both IMV and noninvasive positive pressure ventilation (NPPV) in patients with HM admitted to the ICU. Their results revealed that the ICU mortality rate in the IMV group was three times as high as that in the NPPV group (100 vs. 37%). Overall, NIMV has significantly decreased the risk of death by obviating the need for endotracheal MV in critically ill HM patients. On the other hand, the higher mortality after NIMV failure is in line with studies of patients with HMs (Molina et al. 2012; Gristina et al. 2011; Azoulay et al. 2014), which calls for a more pragmatic approach towards the use of NIMV (Soares et al. 2010a). According to a previous study, first-line NIMV might be deleterious in patients in which the ARDS criteria has been met (Adda et al. 2008). The timely and earlier utilization of NIMV has been recommended, as it can reduce the likelihood of intubation (Hilbert et al. 2001b), ventilator-associated lung injury (Azoulay et al. 2001), and pneumonia or sepsis (Rabbat et al. 2005). In addition, the trials using high-flow nasal oxygen therapy (Mokart et al. 2015a) and extracorporeal gas exchange (Mokart et al. 2015b) have shown that these modalities could be promising alternative approaches to mechanical ventilation in HM patients who also have severe acute respiratory failure.

The utilization of IMV is one of the most powerful predictors for predicting the outcome for patients with HM admitted to the ICU. The timely and appropriate use of NIMV can prevent the deterioration of respiratory failure, thus avoiding the need for patient intubation.

#### Organ failure

Organ failure is a powerful prognostic factor in non-cancer patients admitted to the ICU (Namendys-Silva et al. 2015; Leone et al. 2015). Similarly, critically ill patients with HM admitted to ICU often suffer from multi-organ failure, which also plays an important role in their prognosis (Hampshire et al. 2009; Bird et al. 2012; Gordon et al. 2005; Azoulay et al. 1999; Afessa et al. 1992). In an earlier study, only 1 of the 12 HM patients admitted to the ICU with multi-organ failure survived to discharge, and the authors proposed that HM patients with inadequate function in two or more organs are unable to benefit from intensive care (Brunet et al. 1990). A more recent prospective, multicenter cohort study confirmed that poor outcome was significantly associated with organ failure (Azoulay et al. 2013). Moreover, the number of dysfunctional organs appears to have even more powerful predictive value (Hampshire et al. 2009; Bird et al. 2012; Evison et al. 2001; Namendys-Silva et al. 2013). In another retrospective study performed in Switzerland, the mortality rates of HM patients by day 60 after ICU admission were 16, 36, 64, and 83% for zero, one, two, or three failing organs, respectively (*P* < 0.0002) (Evison et al. 2001).

These data suggest that the early recognition and management of organ failure can lead to more prompt ICU admission and therefore, better survival for patients with HM.

### Scoring systems for overall wellness

Scoring systems have been proven to be of important value for estimating the risk of death and for evaluating the severity of the acute organ failure, especially in patients with HM, but were of less useful for predicting individual prognosis (Schellongowski et al. 2004). There is consensus that severity-of-illness scoring systems perform equal or even better than some other prognostic factors, such as underlying malignancies or disease status. Geerse et al. (2011) showed that the ICU mortality of critically ill HM patients was related to multiple organ failure and the need for mechanical ventilation or inotropic/vasopressor therapy. Therefore, the scoring systems should be considered as useful measures of the patient’s severity of acute illness.

Commonly used scoring systems include the following: Acute Physiology and Chronic Health Evaluation (APACHE and APACHE II), Sequential Organ Failure Assessment (SOFA), and Simplified Acute Physiology Score (SAPS) (Schellongowski et al. 2004; Sawicka et al. 2014). Among these, SOFA is deemed to be the most frequently used system in HM patients admitted to the ICU (Geerse et al. 2011; Lamia et al. 2006; Merz et al. 2008). SOFA was initially devised in 1994 to assess the prognosis of patients with sepsis and septic shock (Vincent et al. 1996), and it has been widely used in ICU patients in recent years. Various forms of SOFA scores, which are taken at different points during the admission or ICU stay, have been used (Cornet et al. 2005; Soares et al. 2010b). In one study, SOFA score ≥15 was associated with 100% ICU mortality in HM patients (Cornet et al. 2005). Additionally, another large prospective multicenter study that included 50 HM patients (7% of all subjects) showed that high SOFA scores (OR 1.25; 95% CI 1.17–1.34) were associated with severe organ failure and increased hospital mortality (Soares et al. 2010b).

Several other studies assessed the utility of monitoring the trend of SOFA scores over time to predict the mortality of HM patients admitted to ICU (Geerse et al. 2011; Liu et al. 2015). For example, Geerse et al. (2011) reported that patients with decreased SOFA scores through the course of ICU admission had significantly better prognosis than patients with unchanged or increasing SOFA scores. Our group replicated this result, and showed that the trend of the SOFA scores within the first 48 h of ICU admission had the most significant prognostic power (Liu et al. 2015).

The usefulness of APACHE II in HM patients is more controversial among the research literature. Some results have found its value for predicting mortality (Hampshire et al. 2009; Owczuk et al. 2005; Rabbat et al. 2005; Yeo et al. 2012; Parakh et al. 2014), while others have not (Lloyd-Thomas et al. 1988; Hampshire et al. 2009; Afessa et al. 1992; Sawicka et al. 2014). Our previous study confirmed the important value of APACHE II, suggesting that this score system may aid in evaluating HM patients before and after ICU transfer in China (Liu et al. 2015).

The SAPS II scoring system was developed to gauge the risk of hospital mortality for ICU patients (Minne et al. 2012; Vosylius et al. 2004). Sawicka et al. (2014) evaluated different scoring systems, including APACHE II, SAPS II and SOFA. Their statistical analysis showed SAPS II score was the only independent risk factor of patient death in multivariate analysis (Sawicka et al. 2014).

Although all scoring systems provide an estimate of the severity of organ failure and acute illness in HM patients, none yields adequately comprehensive and reliable information to be used alone. In addition, the scoring systems have only been used in retrospective or cohort studies with relatively small numbers of HM patients and were conducted at single academic institutions. Their usefulness needs to be yet confirmed in large prospective studies.

### Hematopoietic stem cell transplantation (HSCT)

For selected patients with HM, stem cell transplant can provide long-lasting remission. After allogeneic HSCT, some patients inevitably require intensive care. However, the treatment and health care of general HM patients deviates significantly from HSCT patients, due to high doses of chemotherapy, severe immunosuppression, and isolation. The ICU mortality for HSCT patients is generally high, at 33–65% (Soubani et al. 2004; Bruennler et al. 2007; Afessa et al. 2003; Pene et al. 2006). In recent years, the development of targeted therapy, careful patient selection, and more attentive post-transplant nursing, has substantially improved the outcome of these patients (Bird et al. 2012). A large retrospective cohort study performed by Huynh et al. (2009) showed that the outcome of HSCT patients who required ICU-level care was not as poor as previously described, and the authors observed an improvement in the survival of HSCT patients who required mechanical ventilation. However, the outcome still remains poor for HSCT recipients who develop severe graft versus host disease (GVHD) or other severe complications (Schellongowski et al. 2004; Sawicka et al. 2014). One study noted high mortality rates of up to 100% in ICU patients who developed multiple organ failures after bone marrow transplantation. However, the size of the subgroup in this study was too small for this observation to reach significance (Afessa et al. 1992). In a large study of 398 patients of HM admitted to ICU after HSCT, the morality rate was observed to be 100% if the patients developed acute lung injury, received more than 4 h of pressors, or had sustained hepatic and renal failure (Rubenfeld and Crawford 1996). On the other hand, HSCT patients who do not have uncontrolled GVHD and remain on functional mechanical ventilation have been shown to have more optimistic survival rates (Pene et al. 2006; Gooley et al. 2010; Price et al. 1998). Thus, HSCT recipients without severe organ failure or GVHD would likely benefit from intensive care. Knowledge deficits among ICU physicians on the caring of HSCT recipients may negatively affect the outcome as well. Accordingly, timely communication and close collaboration between hematologists and intensivists could improve patient care. In addition, future studies should compare the prognosis of patients who had undergone different types of HSCT.

## Controversial prognostic factors

### Type and disease status of HM

HM is a broad category of diagnoses, which include both acute and chronic leukemias, various types of lymphomas, and dysfunctional hematopoiesis such as aplastic anemia. The disease statuses include newly diagnosed, remission, and relapsed or progression. Some previous studies have noted that both the type and status of disease were independent prognostic factors for survival. For instance, the ICU mortality rate was lower for HM patients who were in remission status than those with progressive disease (Azoulay et al. 2013; Aygencel et al. 2014). Moreover, ICU mortality was significantly higher in patients diagnosed with acute leukemia (Yeo et al. 2012; Groeger et al. 1999). For example, in one study, the ICU mortality rate of patients diagnosed with acute lymphatic leukemia (ALL) was as high as 100%, although the sample size was too small to make a scientific conclusion (Geerse et al. 2011).

With improvements in supportive care and unbiased patient selection, an increasing number of publications have revealed that the nature of HM does not influence patient prognosis (Lamia et al. 2006; Benoit et al. 2003; Merz et al. 2008; Bruennler et al. 2007). Some studies have demonstrated that patients with de novo acute myeloid leukemia or diffuse large B cell lymphoma show good long term prognosis if they survive the acute disease leading to ICU admission (Schellongowski et al. 2011; Wohlfarth et al. 2016). In a retrospective study, the rate of ICU discharge was 48.8% in remission patients and 38.2% in active/progressive patients, which was not statistically significant (Benoit et al. 2003). A separate study showed that underlying malignancies determined long-term survival, but did not predict ICU or hospital mortality (Massion et al. 2002). Thus, whether the nature of the disease can significantly affect the prognosis of HM patients admitted to the ICU remains debatable. In clinical practice, type and status of HM should not preclude ICU admission in patients who may otherwise benefit from intensive care.

### Infection

Infection is a common reason for ICU admission, especially for critically ill patients (Schuster and Marion 1983; Schellongowski et al. 2004). HM patients are more vulnerable to infection than other patients because of the nature of immunosuppression, medications, particularly steroids, chemotherapy, and stem cell transplant (Hollis 1996). It was previously observed that the incidence of Gram-negative sepsis increased annually in patients with HM (Soubani et al. 2004). The study performed by Hampshire et al. showed that the ICU mortality rate for HM patients suffering from severe infection reached 66.6%, and infection was independently associated with high hospital mortality. The ICU mortality rate was even higher in HM patients with invasive fungal infection (Bird et al. 2012; Sipsas and Kontoyiannis 2012), and reached 79% in a longitudinal observation study (Bird et al. 2012).

However, existing research has not reached a definitive conclusion on how infection affects survival. Some studies actually showed that HM patients had better outcomes if the ICU admission was precipitated by bacteremia (Benoit et al. 2003, 2005; Depuydt et al. 2004, 2010). One explanation may be that bacteremia leads to prompt ICU admission and prevents the development of severe sepsis or septic shock. Variations in the definition of infection may also change its association with prognosis. Confirmed infection is usually defined as positive culture from a sterile site, but the diagnosis of some infections, such as pneumonia, often depends on clinical signs and radiologic judgments, which may not be accurate (Benoit et al. 2003; Reyes et al. 1999). Nevertheless, a broader ICU admission policy may be beneficial to patients with bacteremia.

### Neutropenia

Neutropenia is generally defined as an absolute neutrophil count less than 1000/μL. The significance of neutropenia as a risk factor for HM patients at the time of admission to ICU or during the ICU stay remains controversial. Some publications have reported higher mortality in neutropenic patients, especially when mechanical ventilation is essential (Khwankeaw and Bhurayanontachai 2014). In a retrospective observational study, neutropenia was independently associated with poor outcomes (Benoit et al. 2003). The higher mortality in this subgroup may be attributed to an increased susceptibility to nosocomial infections and severe sepsis (Benoit et al. 2003).

On the contrary, other researchers reported that neutropenia was not independently associated with higher mortality in HM patients admitted to the ICU (Liu et al. 2015). In our previous analysis, neutropenia at the time of admission was not a prognostic factor for ICU mortality (Liu et al. 2015). Interestingly, in another retrospective cohort study, mortality was not significantly higher, even when it persisted throughout the ICU stay or had been present for more than 21 days prior to admission (Geerse et al. 2011). In these cases, the contribution of neutropenia may have been masked by the severe multi-organ failure these patients also suffered from (Blot et al. 1997).

### Studies on HM patients from Asian countries

In the last decade, the ICU mortality rates for HM patients dropped to 26–56% in most of the western countries (Geerse et al. 2011; Evison et al. 2001; Benoit et al. 2003; Kroschinsky et al. 2002; Rabbat et al. 2005; McCaughey et al. 2013), although the mortality still remained higher for post HSCT patients (Townsend et al. 2013). A study from Poland reported an ICU mortality of 75.7%, which was much higher than the overall Western standard, and may be attributed to a comparably higher proportion of patients with mechanical ventilation (Sawicka et al. 2014). Presently, we could not find any studies published from African countries. Of most importance, studies published from Asian countries (China, Korean, India and so on) have revealed that the ICU mortality rate in these countries is higher than that in most western countries, ranging from 55.2 to 84.1% (Liu et al. 2015; Khwankeaw and Bhurayanontachai 2014; Metan et al. 2013; Bahammam et al. 2005; Park et al. 2011; Bajwa et al. 2010). More surprisingly, the highest hospital mortality was shown to be in Korea (84.1%), where the access to advanced medical technology is relatively unrestricted (Yeo et al. 2012).

How can we explain the comparably high mortality rate in Asian countries? First, patients with HM admitted to the ICU in Asian countries may severely suffer from more organ failure and acute illness. For instance, the Asian studies had higher proportions of patients who required IMV or vasopressors (64% required IMV in Turkey and 64.5% required IMV and 67.8% required vasopressors in China). A greater number of patients also had acute leukemia and other acute malignancies, which are associated with a high risk for treatment-related complications (Liu et al. 2015; Yeo et al. 2012). Second, due to insufficient intensive care resources and high population densities, the more stringent ICU admission policies in these countries may have resulted in delayed care (Murthy et al. 2015). For instance, only 0.61% of patients with HM were transferred to the ICU in our previous study involving a three-hospital academic center in China, which is much lower than what was reported in a study from Brazil (5.9%) (Liu et al. 2015; Lecuyer et al. 2007). Additionally, the occupancy of ICU beds in Asian countries is far less than that of western countries (Liu et al. 2015). Previous studies have also shown that ICU centers with high volumes of patients with HM had significantly decreased mortality (Zuber et al. 2012; Lecuyer et al. 2008). Many researchers have suggested that a more reasonable goal is the early identification of the subgroup of patients whose probability of survival is very low despite advanced ICU support (Khwankeaw and Bhurayanontachai 2014). Moreover, the relatively high ratio of mortality to incidence in Asian countries may reflect different cultural beliefs. According to a large scale statistical analysis, 19% of patients in China who would otherwise deteriorate in the ICU were likely to be discharged home near the end of life, while in Brazil, this ratio was as high as 75% (Broad et al. 2013).

Therefore, the high ICU mortality rate may be stem from a combination of the study population characteristics, the distribution of medical resources, and cultural differences on the end of life care. Most of the current data has come from early or retrospective studies, and further prospective and large multi-centered studies should test these hypotheses in Asian regions.

### ICU admission policy

Most countries have relevant criteria regarding ICU admission for critically ill patients, and this is usually based on the need for treatments for organ failure, the frequency of monitoring for vital signs and the need for intensive nursing care (Liu et al. 2015). In most cases, the decision to shift to the ICU depends on an agreement between the specialist physicians and family members, which introduces subjective and emotional factors (Hill 2010). Because of family or patient wishes, some patients with excellent prognosis may be excluded from intensive care and vice versa. The majority of studies that we reviewed recommended broader ICU admission criteria for HM patients to allow more patients to benefit from the higher level of care (Hill 2010; Hampshire et al. 2009; Aygencel et al. 2014; Malak et al. 2014; Azoulay et al. 2011; Thiery et al. 2005). The milestone of the “ICU trial” study performed by Lecuyer et al. (2007) recommended that we should provide an alternative to ICU refusal in patients with malignancies for which potentially life-supporting treatments are available for improving their survival rates. In another large, prospective, multicenter cohort study, less than 24 h between presentation and ICU admission was associated with better hospital survival (Azoulay et al. 2013). Similarly, one study also revealed that the time elapsed between hospital and ICU admission was positively correlated with ICU mortality (Hampshire et al. 2009). Consequently, broader admission policies can facilitate the admission of patients from the emergency departments to the ICU and the stepping-up of floor patients, thereby preventing treatment delay for critically ill HM patients with a high chance of deterioration. The prompt ICU admission of HM patients with acute respiratory failure or bacteremia could decrease the risk of intubation and septic shock (Depuydt et al. 2010). Conversely, delayed admission and suboptimal treatment may lead to the poor outcome in the ICU. In a large single-center study of HM patients admitted to the ICU in London, the ICU, in-hospital, and 6-month mortalities were 33.7, 45.7 and 59.3%, respectively. The authors attributed the lower ICU mortality to the early and prompt admission of patients at high risk for organ failure based on the senior hematologists’ evaluation and the expert cancer care in the ICU (Bird et al. 2012). The insight from this study suggest that specialist ICUs dedicated to the care of cancer patients may help increase the access of HM patients to ICUs and improve their prognosis. In summary, the existing evidence suggests that HM patients with single organ failure should be promptly admitted to the ICU and that a close collaboration between hematologists and ICU specialists is beneficial for patient care (Table 1).

**Table 1:** Summary of selected studies mentioned in this article

References	Country	Region	First author	Patients and sample size	Significant prognostic factors	Organ failure	Scoring systems	ICU mortality (%)	Hospital mortality (%)	6-month mortality (%)
Lloyd-Thomas et al. (1988)	UK	Europe	Lloyd-Thomas	HM N = 60	The dysfunction of an increasing number of organ systems, an APACHE II score of greater than 30, failure of the malignancy to respond to chemotherapy, and persistent leucopenia all			78		
Yau et al. (1991)	UK	Europe	Yaul	HM N = 92	The nature and progress of the underlying malignancy		APACHE II: 25.9		77	
Evison et al.(2001)	Switzerland	Europe	Evison	HM N = 78	Multi-organ failure and evidence of liver damage	47% required vasopressors; 43% required MV	APACHE II:18	26		
Kroschinsky et al. (2002)	Germany	Europe	Kroschinsky	HM N = 104	mechanical ventilation, SAPS II	52% required MV	SAPS II: 46	44		63
Benoit et al. (2003)	Belgium	Europe	Benoit	HM N = 124	Leukopenia, vasopressors, urea of >0.75 g/L at admission, recent bacteremia	54% required ventilation; 54% required pressors	APACHE II: 26 ± 7.7; SAPS II: 53 ± 17.8	42	54	66
Rabbat et al. (2005)	France	Europe	Rabbat	HM N = 83	Simplified acute physiology score II and need for IMV	47% required IMV	SAPS II 54.9 ± 26.7	34		
Lamia et al. (2006)	France	Europe	Lamia	HM N = 92	Severity and three organ failure scores on day 1 and Delta scores	41% required IMV; 50% required pressors	SOFA: 9 ± 5 SAPS II:60 ± 22		58	
Verplancke et al. (2008)	Belgium	Europe	Verplancke	HM N = 352	–	49% Required IMV; 41% required pressors	APACHE II: 24.5 ± 7.4		54.4	
Ferrà et al. (2008)	Spain	Europe	Ferrà	HM N = 116	Need of mechanical ventilation, cardiovascular vasoactive drugs			54		
Merz et al. (2008)	Australia	Oceania	Merz	HM N = 101	SAPS II at ICU admission, Mechanical ventilation, Renal replacement therapy, ICU length of stay	55.3% required MV		33.7		
Hampshire et al. (2009)	UK	Europe	Hampshire	HM N = 7689	Bone marrow transplant, Hodgkin’s lymphoma, severe sepsis, age, length of hospital stay prior to intensive care admission, tachycardia, low systolic blood pressuretachypnoea, low Glasgow Coma Score, sedation, PaO2:FiO2, acidaemia, alkalaemia, oliguria, hyponatraemia, hypernatraemia, low haematocrit, and uraemia		APACHE II: 24.4 ± 7.9	43.1	59.2	
Geerse et al. (2011)	Netherland	Europe	Geerse	HM N = 86	Organ failure, a need for mechanical ventilation or inotropic/vasopressor therapy	52% required IMV, 52% required pressors	APACHE II:28.8 ± 7.9 SOFA:10.1 ± 3.4	56	65	
Turkoglu et al. (2011)	Turkey	Asia	Turkoglu	HM N = 128	Low Glasgow coma scale, prior immunosuppressive treatment, neutropenia, IMV, and severe sepsis	64% required IMV	APACHE II:24	65	70	
Bird et al. (2012)	UK	Europe	Bird	HM N = 199	Mechanical ventilation;≥2 organ failures	51.9% required IMV; 51.5% required pressors	APACHE II:21	33.7	45.7	59.3
Yeo et al. (2012)	South Korea	Asia	Yeo	HM N = 227	Acute leukemia, need for invasive mechanical ventilator, use of inotropic/vasopressor agents, and Acute Physiology, Chronic Health Evaluation II scores	48% required IMV; 54.6% required inotropes	APACHE II: 19.4 ± 0.5	84.1	89.9	
Namendys-Silva et al. (2013)	Mexico	America	Namendys-Silva	HM N = 102	Neutropenia at the time of ICU admission, the need for vasopressors, need for invasive mechanical ventilation, serum creatinine > 106 μmol/L	85.3% required IMV; 77.5% required pressors	SOFA: 9.8 ± 4; APACHE:17.5 ± 6; SAPS II: 42.5 ± 15.3	46.1	57.8	
Hill et al. (2013)	UK	Europe	Hill	HM N = 147	Culture proven infection, age, MV and inotropes			56	79	
McCaughey et al. (2013)	UK	Europe	McCaughey	HM N = 21	(APACHE II) scores, and decreased requirements for invasive ventilation and inotropic support		APACHE II:23	43		67
Azoulay et al. (2013)	France	Europe	Azoulay	HM N = 1011	Cancer remission; time to ICU admission less than 24 h, poor performance status, Charlson comorbidity index, allogeneic HSCT, organ dysfunction score, cardiac arrest, acute respiratory failure, malignant organ infiltration, invasive aspergillosis	47.9% required IMV, 51.2% required pressors			60.7	
Townsend et al. (2013)	UK	Europe	Townsend	HM allo-HSCT N = 213	The need for ventilation	50% required MV;45% required pressors	APACHE II: 23	68.0		
Sawicka et al. (2014)	Poland	Europe	Sawicka	HM N = 99	SAPS II			75.7		
Khwankeaw and Bhurayanontachai (2014)	Thailand	Asia	Khwankeaw	HM N = 145	Mechanical ventilation, the use of vasopressors and the APACHE II scores			55.2		
Liu et al. (2015)	China	Asia	Liu and Cheng	HM N = 121	The use of IMV, APACH II at admission, SOFA trend	64.5% required IMV, 67.8% required pressors	APACHE II:20.84 ± 0.89 SOFA:10.75 ± 0.48	60.3		90.9

## Conclusions

Many studies have investigated the prognostic factors for HM patients admitted to the ICU. Some factors identified in the earlier studies, such as age, underlying diagnosis of malignancy, and disease status, were proven to be irrelevant in more recent studies. The role of other clinical factors, such as infection and neutropenia, remain controversial. Although the incorporation of comprehensive scoring systems for assessing overall health and organ failure status have helped clinicians to estimate prognosis, none is specific enough for predicting mortality in critically ill patients with HM.

The overall ICU mortality rate for HM patients ranges between 26 and 84.1%, and differs by study population and region. The mortality rate is comparably higher in Asian countries (Yeo et al. 2012). Broader ICU admission policies are recommended to ensure earlier treatment and for improving mortality. The admission of HM patients to the ICU raises ethical concerns for both the hematological and intensive care teams. The decision of transfer to the ICU has major consequences on end of life care for both the patients and their relatives. It affects the distribution of medical resources, human resources, and the organizational and economic aspects for the ICU and global health policy. A guiding principle for ICU admission is that it should be reserved for patients with reversible medical conditions and a reasonable chance of substantial recovery. Thus, the basis of intensive care medicine is optimizing the patient’s physiology by delivering supportive therapy, while attempting to treat the underlying disease. With more scientific patient selection and unbiased judgment, the prognosis of HM patients admitted to the ICU may improve.
